# The MCL1-specific inhibitor S63845 acts synergistically with venetoclax/ABT-199 to induce apoptosis in T-cell acute lymphoblastic leukemia cells

**DOI:** 10.1038/s41375-018-0201-2

**Published:** 2018-07-15

**Authors:** Zhaodong Li, Shuning He, A. Thomas Look

**Affiliations:** 1 Department of Paediatric Oncology, Dana-Farber Cancer Institute, Harvard Medical School, Boston, MA 02215 USA; 2 Division of Haematology/Oncology, Boston Children’s Hospital, Boston, MA 02215 USA

**Keywords:** Acute lymphocytic leukaemia, Cancer therapy

The mitochondrial cell death pathway is initiated by pro-apoptotic BH3-only effector proteins, such as BIM, BID, NOXA, and PUMA, which activate the multidomain cell death proteins, BAX and BAK [1]. The survival of tumor cells, as well as normal cells, is promoted by anti-apoptotic BCL-2-family members, including BCL-2, BCL-X_L_, and MCL1, which bind and sequester BH3-only proteins, thus preventing them from activating BAX and BAK [1]. Cancer cells tend to rely more heavily on anti-apoptotic BCL-2 family proteins because of replicative and other stresses that accompany malignant transformation, and thus are “primed” to undergo apoptosis more readily than normal cells [1]. Small molecules have been developed that mimic the BH3 domain and block binding of endogenous BH3 proteins to a groove on the surface of one or more of the pro-survival proteins. Promising examples are navitoclax/ABT-263, targeting BCL-2, BCL-X_L_, and BCL-W, and more recently venetoclax/ABT-199 targeting BCL-2 alone [2]. The most successful of these drugs is the BCL-2 inhibitor venetoclax, which is approved for the treatment of chronic lymphocytic leukemia (CLL) [2, 3] and has shown considerable activity in therapy for other cancers, such as acute myeloid leukemia (AML) [4]. Venetoclax is better tolerated than navitoclax, because it does not bind to BCL-X_L_, which is required for the survival of normal platelets [3].

Although T-cell acute lymphoblastic leukemia (T-ALL) is similar in many ways to CLL and AML, it has not responded well to venetoclax or navitoclax BH3 mimetics, presumably because it expresses active pro-survival proteins other than BCL-2, BCL-X_L_, and BCL-W. We and others have previously tested venetoclax and navitoclax against a number of human T-ALL cell lines, observing submicromolar activity only in the Loucy cell line, which is thought to represent early thymocyte precursor (or ETP) ALL, a T-ALL subtype with a particularly poor prognosis [5–7]. Venetoclax has been tested in multiple clinical trials (https://clinicaltrials.gov/) and is approved for the treatment of CLL [2, 3], but it has just begun to be tested in patients with T-ALL. This led us to postulate that T-ALL cells might depend upon MCL1, a labile pro-survival member of the BCL-2 family that has been shown to mediate resistance to BCL-2 inhibitors [8, 9]. Thus, inhibitors of MCL1 appear especially attractive for combination with BCL-2 inhibitors for the treatment of T-ALL and other cancers. A new BH3 mimetic, S63845, was recently found to selectively target MCL1, and S63845 has been tested in many preclinical models of human cancer [10], including breast cancer [11], but not in T-ALL. Clinical data for the activity of MCL1 inhibitors, including S63845, have as yet not been reported. Thus, we sought to test the hypothesis that S63845 will actively induce apoptosis in T-ALL cells when given as a single agent, and also that it might produce synergistic effects when used in combination with the BCL-2 inhibitor venetoclax.

Thus, we first tested a panel of 11 T-ALL cell lines for their sensitivity to S63845. Each line was sensitive to S63845 treatment as shown by 50% growth inhibitory (IC_50_) values in a submicromolar range (Fig. 1a). The values for two of the most sensitive cell lines, MOLT-3 and RPMI-8402, were as low as 10 nM. These results indicate that MCL1 plays an important role in maintaining the survival of most T-ALL cells. Consistent with previous studies in AML, chronic myeloid leukemia, and diffuse large B-cell lymphoma cell lines [10], we did not observe correlation between MCL1 protein levels and sensitivity to S63845 in these T-ALL cell lines. Similarly, BCL-2 and BCL-X_L_ levels did not predict response to S63845 treatment (Supplementary Figure 1). We also tested the activity of A-1210477, another MCL1-specific inhibitor [12], against these T-ALL cell lines in comparison with the activity of S63845. The IC_50_ values for A-1210477 were in the high micromolar range (Supplementary Figure 2), indicating that S63845 is much more potent in a cellular context, even though both drugs exhibit high affinity and specificity for the MCL1 protein in vitro [10, 12].

**Fig. 1:**
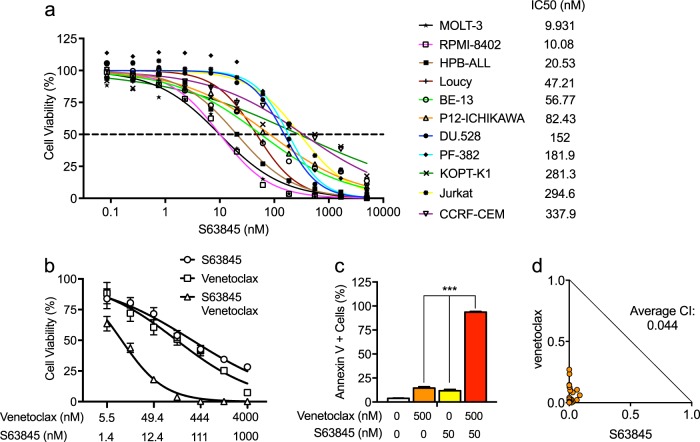
The MCL1-specific inhibitor S63845 actively kills human T-ALL cells and strongly synergizes with venetoclax. **a** Eleven human T-ALL cell lines were treated with serial dilutions of S63845. Cell viability was determined by Cell-Titer Glo assay after 72 h of treatment. **b** KOPT-K1 cells were treated with serial dilutions of venetoclax or S63845, alone or in combination. Cell viability was determined using Cell-Titer Glo after 48 h of treatment. **c** KOPT-K1 cells were treated with 500 nM venetoclax and 50 nM S63845, alone or in combination. Apoptosis was measured by annexin V/PI staining after 24 h of treatment. **d** Combination treatment of venetoclax and S63845 depicted as normalized isobolograms shows strong synergy between the two BH3 mimetics in KOPT-K1 cells. Calcusyn software was used to analyze combination data to produce the isobolograms normalized to the IC_50_ of each drug. The cells were treated with the following serial dilutions of combination doses: venetoclax, from 5.5 nM to 4000 nM; and S63845, from 1.4 nM to 1000 nM for 48 h. A combination index (CI) of 1 indicates an additive effect, CI < 1 a synergistic effect and CI > 1 antagonism

Next, we tested whether the effects of S63845 were mediated through the activation of apoptosis. Using four relatively sensitive T-ALL cell lines based on IC_50_ values—HPB-ALL, Loucy, MOLT-3, and RPMI-8402—we demonstrated the induction of significant apoptosis by annexin V/propidium iodide (PI) staining after 24 h of treatment with S63845 (Supplementary Figure 3a, b). Western blot analysis after 24 h of treatment with 200 nM S63845 showed appreciable PARP cleavage, a reliable marker of cell apoptosis, in each of these four cell lines (Supplementary Figure 3c). Taken together, these data demonstrate that a representative panel of T-ALL cell lines are sensitive to the S63845 inhibitor and thus depend on MCL1 for cell survival.

The anti-apoptotic BCL-2 family proteins BCL-2, BCL-X_L_, and MCL1 can each bind and sequester BH3-only pro-apoptotic proteins, such as BIM, BID, and PUMA [2, 13], raising the possibility that two very specific inhibitors such as venetoclax (BCL-2) and S63845 (MCL1) might act synergistically to kill T-ALL cells. To test this hypothesis, we treated T-ALL cells with serial dilutions of S63845 and venetoclax, either alone or in combination. Our results, shown in Fig. 1b and Supplementary Figure 4 for KOPT-K1 and PF-382 cells, indicate much greater decreased cell viability in cells treated with both agents in combination. This result reflects the induction of apoptosis as shown by annexin V and PI staining (Fig. 1c and Supplementary Figure 5). To determine whether the combined effects of S63845 and venetoclax are additive or synergistic, we performed isobologram analysis after 48 h of combination treatment with serial dilutions of S63845 and venetoclax. These agents proved highly synergistic against each of the four T-ALL cell lines tested—KOPT-K1, PF-382, Jurkat, and CCRF-CEM (Fig. 1d and Supplementary Figure 6), with an average combination index (CI) of < 0.25 (a CI of 1 reflects an additive effect, CI < 1 a synergistic effect and CI > 1 indicates antagonism). As shown in Supplementary Figure 7a, protein levels of MCL1 were increased after venetoclax treatment, but venetoclax treatment had little effect on the expression of both BCL-2 and BCL-X_L_. Based on the work of Choudhary et al. [9], increased MCL1 levels are expected and may be due to increased MCL1 protein stability after treatment with venetoclax. As observed previously [10], treatment of S63845 also caused upregulation of MCL1 levels with little effect on expression levels of BCL-2 and BCL-X_L_ (Supplementary Figure 7b). Thus, the compensatory upregulation of MCL1 suggests a molecular basis for both the lack of response of most T-ALL lines to venetoclax treatment [5] and for the synergy between S63845 and venetoclax in these T-ALL cell lines.

To assess the potential toxicity of this combination in normal cells in vivo, a critical concern when testing such agents for possible use in patients, we turned to our zebrafish model of T-ALL [14]. We first added each of the two compounds at graded doses as single agents to the fish water of normal 3-day-old zebrafish embryos. After 4 days of treatment, we determined that the maximum tolerable dose of both S63845 and venetoclax was 10 μM. Developmental defects of multiple organs including the liver, swim bladder, and gastrointestinal tract were observed in embryos treated with 15 μM S63845 (Supplementary Figure 8). However, we found no evidence of toxicity in embryos treated with 10 μM S63845 alone or in combination with 10 μM venetoclax, indicating that the combination is well tolerated at these dosages by healthy tissues in vivo.

To determine the anti-T-ALL activity of S63845 and venetoclax, we harvested green fluorescent protein (GFP)-labeled zebrafish T-ALL cells from 3-month-old *Tg(rag2:Myc; rag2:EGFP)* transgenic zebrafish [14], and intravenously injected these T-ALL cells into 2-day-old zebrafish embryos [15]. One day after injection, embryos bearing GFP-labeled T-ALL cells were treated with S63845, venetoclax or vehicle (dimethyl sulfoxide), each added to the fish water as single agents. After 4 days of treatment, the GFP-labeled T-ALL cells had proliferated and disseminated throughout the recipient embryos that were treated with vehicle, S63845, or venetoclax (Fig. 2a, b). To further investigate why the Myc-driven zebrafish T-ALL cells are insensitive to MCL1 inhibition as a single agent, we treated zebrafish embryos with S63845 and venetoclax, either as single agents or in combination. Then we analyzed lysates of whole embryos by western blotting, and we observed marked compensatory upregulation of both BCL-2 and MCL1 protein levels upon treatment with either drug (Supplementary Figure 9). BCL-2 was not upregulated in response to S63845 in T-ALL cell lines treated in vitro, accounting for the apoptotic response of T-ALL cell lines but not zebrafish thymocytes in vivo to treatment with S63845 as a single agent. There are not sufficient cells in the zebrafish embryos to study only the thymocytes by western blotting, but at least at the whole embryo level, compensatory upregulation appears to explain resistance to treatment with each drug as single agent.

**Fig. 2:**
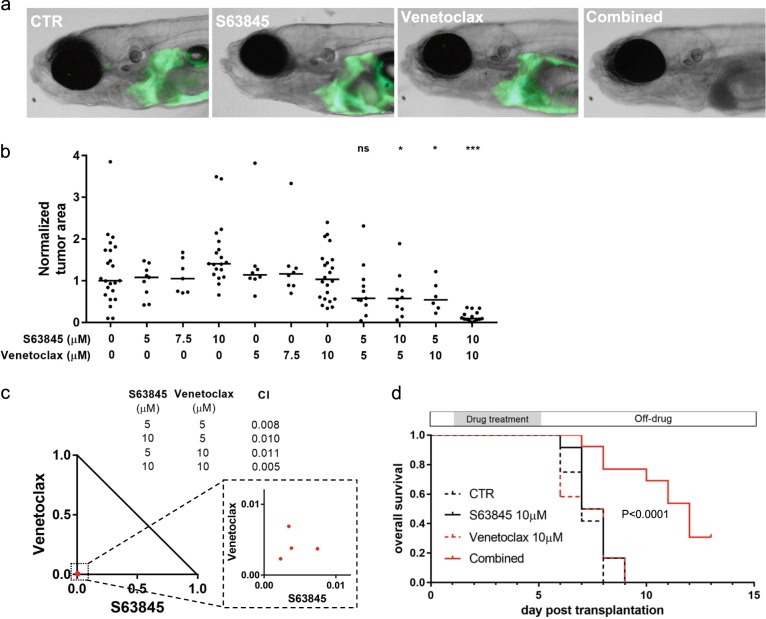
S63845 strongly synergizes with venetoclax in killing T-ALL cells in vivo. **a** Representative zebrafish embryos transplanted with T-ALL cells isolated from *Tg(rag2:Myc; rag2:EGFP)* zebrafish and treated with vehicle, S63845 (10 μM), venetoclax (10 μM), and the combination of S63845 and venetoclax (10 μM of each). **b** Quantification of GFP-positive leukemic area in zebrafish embryos and compared with vehicle treatment with the two-tailed Welch’s *t* test: *P < 0.05; ***P < 0.001. **c** Synergistic effects of S63845 and venetoclax on T-ALL cell kill were analyzed by isobologram analysis. A combination index (CI) of 1 indicates an additive effect, CI < 1 a synergistic effect and CI > 1 indicates antagonism. **d** The combination treatment of S63845 and venetoclax significantly extended the overall survival of T-ALL bearing embryos. Zebrafish embryos transplanted with T-ALL cells were treated as indicated for 4 days, with drug refreshment every 2 days, and then the drugs were removed before feeding was initiated and the developing zebrafish larva were observed for 10 more days (*n* = 12 for CTR, S63845, and venetoclax treatment groups; *n* = 13 for combined treatment group)

The major impact of S63845 and venetoclax was evident when they were administered together. At 10 μM S63845 plus 10 μM venetoclax, this combination greatly reduced the number of leukemic cells in the vast majority of embryos after 4 days of treatment (Fig. 2a, b). Importantly, the synergistic anti-T-ALL effects of S63845 plus venetoclax were documented in vivo at several different dosage levels by isobologram analysis (Fig. 2c), indicating that MCL1 and BCL-2 inhibitors are synergistic at multiple dosage levels leading to T-ALL cell apoptosis in vivo with acceptable tolerance by normal cells.

To address the effects of treatment on survival of the transplanted embryos, we removed the drugs after treating the embryos with S63845 and venetoclax as single agents or combinations for a period of 4 days and then started feeding the embryos and observed them an additional 10 days (Fig. 2d). We found that treatment with the combination of S63845 and venetoclax significantly extended the overall survival of T-ALL bearing embryos, whereas single drug treatment with either drug had no impact survival. The treatments did not affect the viability of leukemia-free fish, excluding possibility that the fish treated with the combination died from toxicity of the treatment they received (Supplementary Figure 10). In addition, the fish treated with the combination exhibited growth of the GFP-labeled T-ALL cells after stopping the drugs and prior to their death. The last four fish were killed at day 13 with rapidly growing, disseminated GFP-positive T-ALL cells for humane reasons (Fig. 2d).

In conclusion, our study demonstrates that the MCL1-specific inhibitor S63845 actively kills human T-ALL cells as a single agent and acts synergistically with venetoclax to more potently induce apoptosis. These drugs were well tolerated in the zebrafish model when given alone and in combination. Interestingly, neither drug alone could effectively kill transplanted T-ALL cells growing in vivo, apparently owing to upregulation of MCL1 and BCL-2 levels in response to treatment with either drug. However, combined administration of S63845 and venetoclax led to profound synergistic activity against T-ALL in vitro and in vivo without appreciable toxicity even at doses that greatly reduced transplanted T-ALL cells in zebrafish embryos. Our results indicate that the newly developed MCL1-specific inhibitor S63845 warrants testing in clinical trials for relapse/refractory patients with T-ALL, both alone and in combination with venetoclax to simultaneously inhibit both MCL1 and BCL-2.

## Electronic supplementary material

Supplementary information

Supplementary information Figure 1

Supplementary information Figure 2

Supplementary information Figure 3

Supplementary information Figure 4

Supplementary information Figure 5

Supplementary information Figure 6

Supplementary information Figure 7

Supplementary information Figure 8

Supplementary information Figure 9

Supplementary information Figure 10
