# Supplementary material for: The MCL1-specific inhibitor S63845 acts synergistically with venetoclax/ABT-199 to induce apoptosis in T-cell acute lymphoblastic leukemia cells
Source: Leukemia. 2018 Jul 15;33(1):262–6. doi: 10.1038/s41375-018-0201-2 (PMC6327051; doi:10.1038/s41375-018-0201-2)
Supplement: Supplementary file 1 — Supplementary information [file 41375_2018_201_MOESM1_ESM.docx]

**SUPPLEMENTARY INFORMATION**

**MATERIALS AND METHODS:**

**Human T-ALL Cell Lines**

The identities of T-ALL cell lines were confirmed by analysis of short tandem repeats using the PowerPlex 1.2 system (Promega) in January 2013, and the T-ALL cell lines used in this study were reconfirmed in February 2016. All T-ALL cell lines were cultured in RPMI-1640 medium supplemented with 10% FBS and penicillin/streptomycin (Invitrogen). Cell lines were tested for mycoplasma contamination and found negative before used for experiments.

**Cell Viability and Apoptosis Analysis**

For viability assay, human T-ALL cells were plated at a density of 20,000 per well in 96-well plates (Corning, Corning, NY, USA) and incubated for indicated times in DMSO (ATCC, Manassas, VA, USA), S63845 (C-1370, Chemgood, Glen Allen, VA, USA), A-1210477 (CT-A121, Chemietek, Indianapolis, IN, USA), and venetoclax/ABT-199 (CT-A199, Chemietek, Indianapolis, IN, USA). Cell viability was determined using Cell-Titer Glo assay (Promega, Madison, WI, USA) and was presented as a relative percentage versus control cells. Apoptosis of human T-ALL cells after treatment was analysed using the BD Annexin V: FITC Apoptosis Detection Kit II (Thermo Fisher Scientific) following manufacturer’s instructions. Cells stained for apoptosis were analysed on a FACScalibur (BD Bioscience, San Jose, CA, USA).

**Western Blotting Analysis**

Whole cell lysates and fish embryos were prepared in RIPA buffer (Cell Signaling, Boston, MA, USA). The primary antibodies included: MCL1 (SC-819, Santa Crutz, Dallas, TX, USA), human BCL-2 (#4223, Cell Signaling, Boston, MA, USA), zebrafish BCL-2 (GTX54185, GeneTex, Irvine, CA, USA), BCL-XL (#2764, Cell Signaling, Boston, MA, USA), PARP (#9542, Cell Signaling, Boston, MA, USA) and anti-β-actin (#4967, Cell Signaling, Boston, MA, USA) antibodies. Secondary antibodies included horseradish peroxidase-conjugated anti-rabbit antibodies (#7074, Cell Signaling, Boston, MA, USA).

**Synergism Analysis of the combination treatment of S63845 and venetoclax/ABT-199 in Human T-ALL Cell Lines**

Human T-ALL cell lines were plated in 96-well plates with 20,000 cells per well and treated with a combination of S63845 and ABT-199 with a range of doses around the IC50 of each drug alone. Cell viability was analysed by Cell-Titer Glo assay (Promega, Madison, WI, USA). Calcusyn software (Biosoft, Cambridge, UK) was used to calculate the potency of drug combination and combination index (CI). Normalized isobolograms were also produced with Calcusyn software.

**Transplantation of Fish Leukemic Cells and Drug Treatment**

All zebrafish studies and maintenance of the animals were performed in accordance with Dana-Farber Cancer Institute IACUC-approved protocol #02-107. For in vivo drug treatment, 3-day-old zebrafish embryos were placed in 48-well plates with 5 embryos per well, and treated with DMSO vehicle, venetoclax and/or S63845 in standard egg water. The treated embryos were checked every day for 5 days and dead embryos were removed on daily basis. For T-ALL transplantation, zebrafish T-ALL cells were harvested by dicing 3-month-old *Tg(rag2:Myc; rag2:EGFP)* transgenic zebrafish in 0.9 x PBS supplemented with 5% FBS. The cell suspension was filtered with Falcon 40-µm cell strainer (Corning, Corning, NY, USA) and loaded into thin-wall borosilicate glass capillary needles (1.0 mm OD, 0.75 mm ID; World Precision Instruments, Sarasota, FL, USA). The recipients, 2-day-old zebrafish Casper embryos, were manually dechorionized and anaesthetized with 0.003% tricaine (Sigma) before being positioned on a 10 cm Petri dish coated with 1% agarose. Intravenous leukaemia transplantation was performed as described (14), with 200~400 cells injected into each recipient. One day later, the 3-day-old recipients were randomly divided into 48-well plates and treated with DMSO vehicle, venetoclax or S63845 in standard egg water for four days, with once drug refreshment at the second day of the treatment. After the four-day treatment, the treated fish were imaged for the fluorescent leukemic cell area in their head area using a Nikon SMZ1500 microscope equipped with a Nikon digital sight DS-U1 camera.

**Statistical Analysis**

Statistical analysis was performed with GraphPad Prism 7.0 software (GraphPad). The quantitative data in Figures 1 are reported as mean values. Two-tailed Student’s t-test with confidence intervals of 95% was used for the analyses. For animal studies in Figure 2b, we used Welch t-test to address the inhomogeneity of variance. With not less than 6 animals per group, we have 95% power to identify leukaemia inhibition, testing at the 0.05 one sided level using Welch’s t-test. For survival analysis in Figure 2e, we used Kaplan-Meier methods and the log-rank test. P-values less than or equal to 0.05 were considered statistically significant.

**SUPPLEMENTARY FIGURE LEGENDS:**

**Supplementary Figure 1.** No correlation was observed between S63845 sensitivity and MCL1, BCL-2 and BCL-X_L_ protein levels in T-ALL cell lines. a) Proteins levels of MCL1, BCL-2 and BCL-X_L_ in T-ALL cell lines determined by western blot. (b-d) Correlation analysis of IC50 values for treatment with S63845 and MCL1 (b), BCL-2 (c) or BCL-X_L_ (d) protein levels.

**Supplementary Figure 2.** 11 T-ALL cell lines were treated with serial dilutions of MCL1-specific inhibitor A-1210477. Cell viability was determined with Cell-Titer Glo after 72h treatment.

**Supplementary Figure 3**. S63845 induced apoptosis in MOLT-3, RPMI-8402, HPB-ALL and Loucy cells. (a) HPB-ALL, Loucy, MOLT-3 and RPMI-8402 cells were treated with DMSO or S63845 as indicated. Annexin V and propidium iodide (PI) staining were performed to determine apoptosis after 24h treatment. (b) The percentages of apoptotic cells were compared with the two-tailed Student’s t-test: *P<0.05; ***P<0.001. (c) HPB-ALL, Loucy, MOLT-3 and RPMI-8402 cells were treated with DMSO or S63845 as indicated. Western blot was performed to determine the PARP cleavage after treatment of 24h.

**Supplementary Figure 4**. PF-382 cells were treated with serial dilutions of venetoclax or S63845, alone or in combination. Cell viability was determined using Cell-Titer Glo after 48 h of treatment.

**Supplementary Figure 5.** (a) KOPTK-1 and PF-382 cells were treated with 500 nM venetoclax and 50 nM S63845, alone or in combination. Apoptosis was measured by annexin V/PI staining after 24h of treatment. (b) The percentages of apoptotic PF-382 cells from (a) were compared with the two-tailed Student’s t-test: ***P<0.001.

**Supplementary Figure 6.** Combination treatment of venetoclax and S63845 depicted as normalized isobolograms shows strong synergy between the two BH3 mimetics in KOPT-K1, PF-382, Jurkat and CCRF-CEM cells. Calcusyn software was used to analyse combination data to produce the isobolograms normalized to the IC50 of each drug. Lower panel is the “expanded scale window” for better view of each data. The cells were treated with the following serial dilutions of combination doses: venetoclax from 5.5nM to 4,000nM and S63845 from 1.4nM to 1,000nM for 48h. Combination index (CI) of 1 indicates an additive effect, CI < 1 a synergistic effect and CI> 1 indicates antagonism.

**Supplementary Figure 7.** (a) Venetoclax treatment resulted in increased MCL1 protein levels in KOPT-K1 and PF-382 cells. KOPT-K1 and PF-382 cells were treated with different doses of venetoclax for 24 h. Total protein lysates were harvested for western blot. (b) S63845 treatment also resulted in increased MCL1 protein levels in KOPT-K1 and PF-382 cells.

**Supplementary Figure 8.** Determination of the maximum tolerated dose (MTD) of S63845. Representative embryos treated for 4 days with S63845, starting from 3 day of age. Embryos treated with 15 μM S63845 exhibited significant developmental defects in multiple organs including the liver (L), swim bladder (S) and gastrointestinal tract (G). One day later, all embryos treated with 15 μM S63845 died, whereas 10 μM S63845 did not induce lethality.

**Supplementary Figure 9.** S63845 (10 μM) and venetoclax (10 μM) increased MCL1 and BCL2 protein levels in zebrafish embryos.

**Supplementary Figure 10.** Kaplan-Meier curves for overall survival indicated that the treatment of venetoclax and S63845, either as single agents or in combination, did not affect the viability of leukaemia-free zebrafish embryos (n=12 for each group).
