# Supplementary figures and images for: The MCL1-specific inhibitor S63845 acts synergistically with venetoclax/ABT-199 to induce apoptosis in T-cell acute lymphoblastic leukemia cells
Source: Leukemia. 2018 Jul 15;33(1):262–6. doi: 10.1038/s41375-018-0201-2 (PMC6327051; doi:10.1038/s41375-018-0201-2)

Supplementary Figure 1

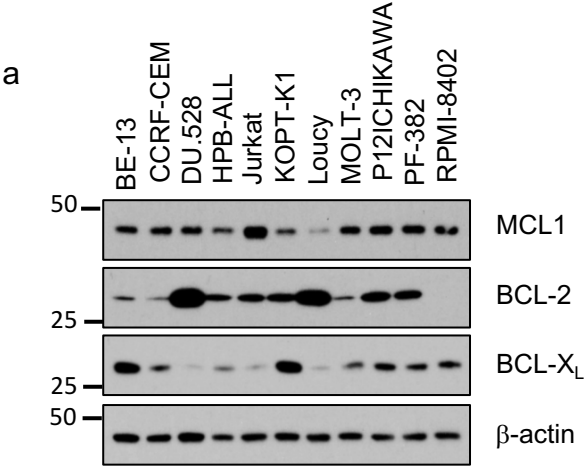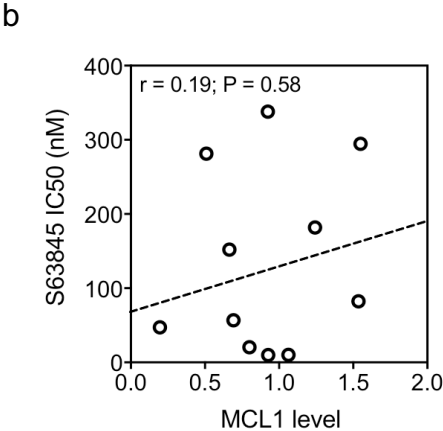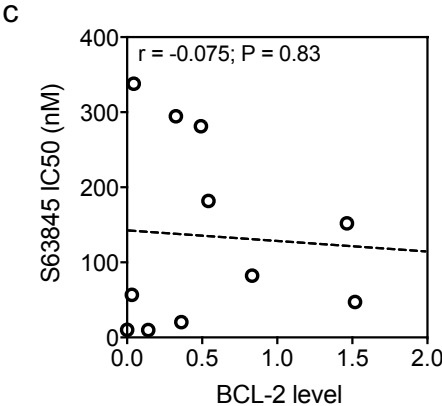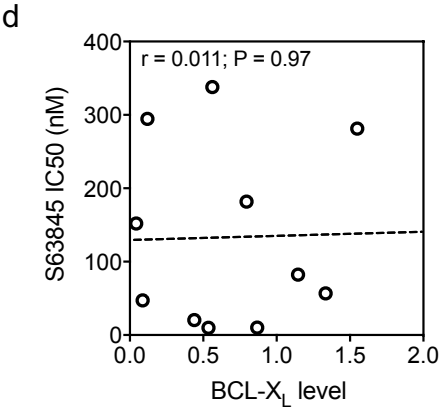

Supplement: Supplementary file 2 — Supplementary information Figure 1 [file 41375_2018_201_MOESM2_ESM.pdf]

Supplementary Figure 2

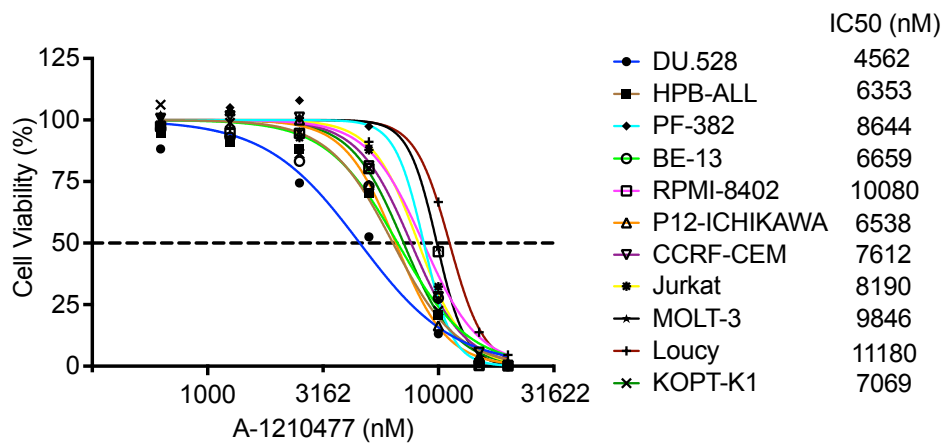

Supplement: Supplementary file 3 — Supplementary information Figure 2 [file 41375_2018_201_MOESM3_ESM.pdf]

# Supplementary Figure 3

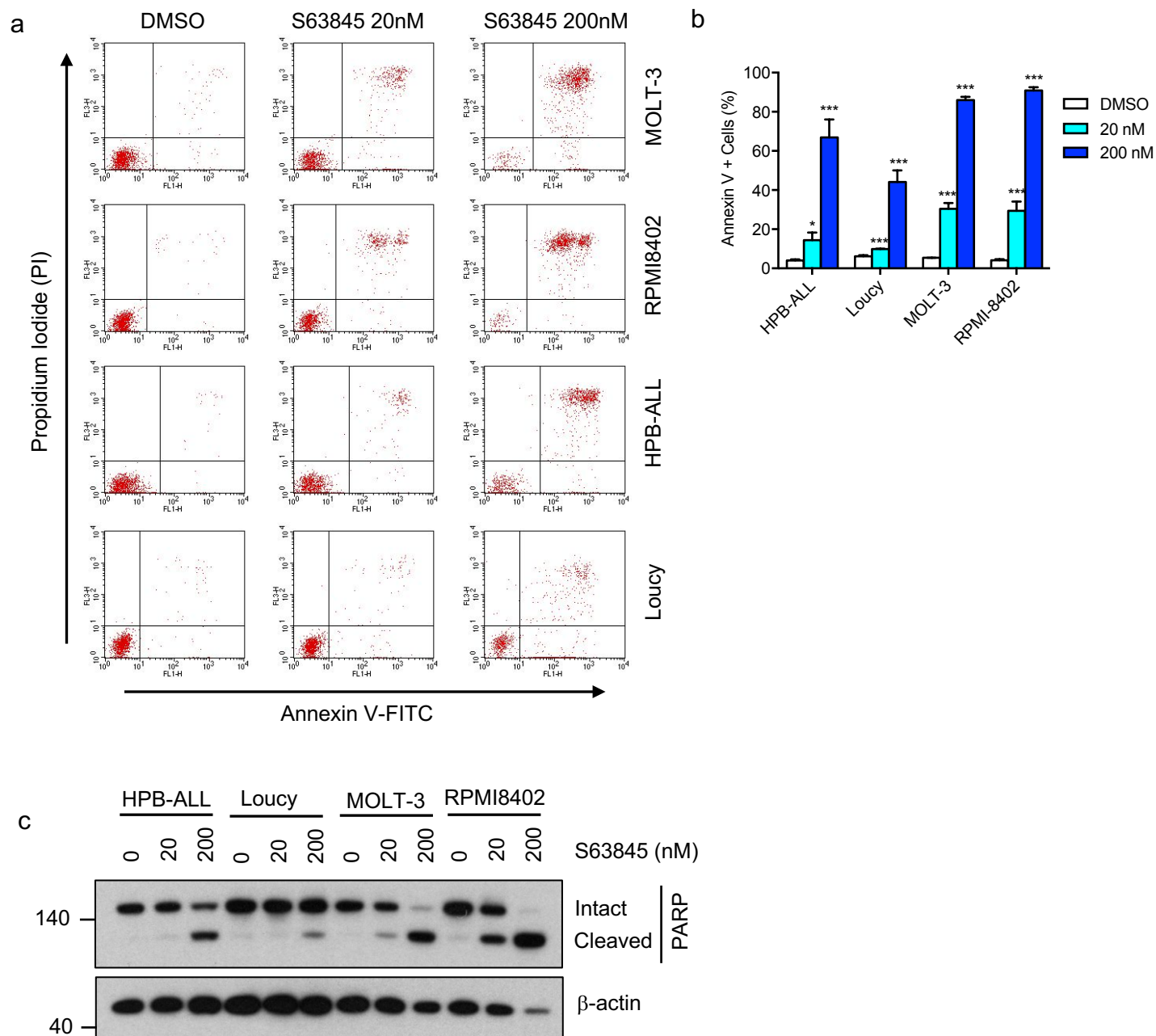

Supplement: Supplementary file 4 — Supplementary information Figure 3 [file 41375_2018_201_MOESM4_ESM.pdf]

Supplementary Figure 4

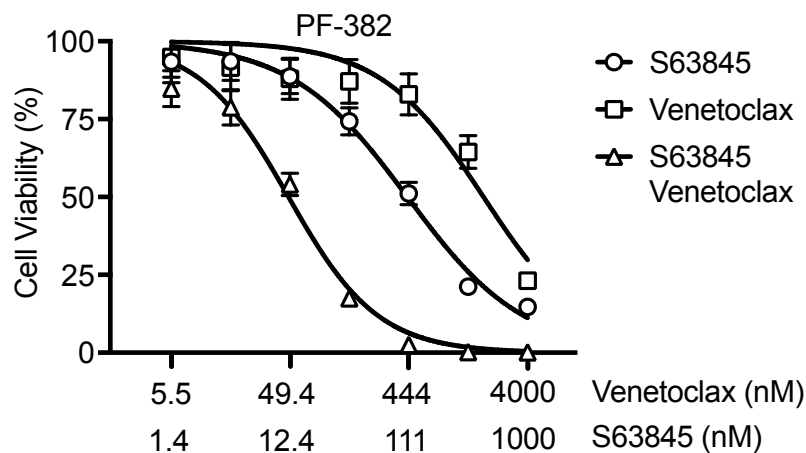

Supplement: Supplementary file 5 — Supplementary information Figure 4 [file 41375_2018_201_MOESM5_ESM.pdf]

Supplementary Figure 5

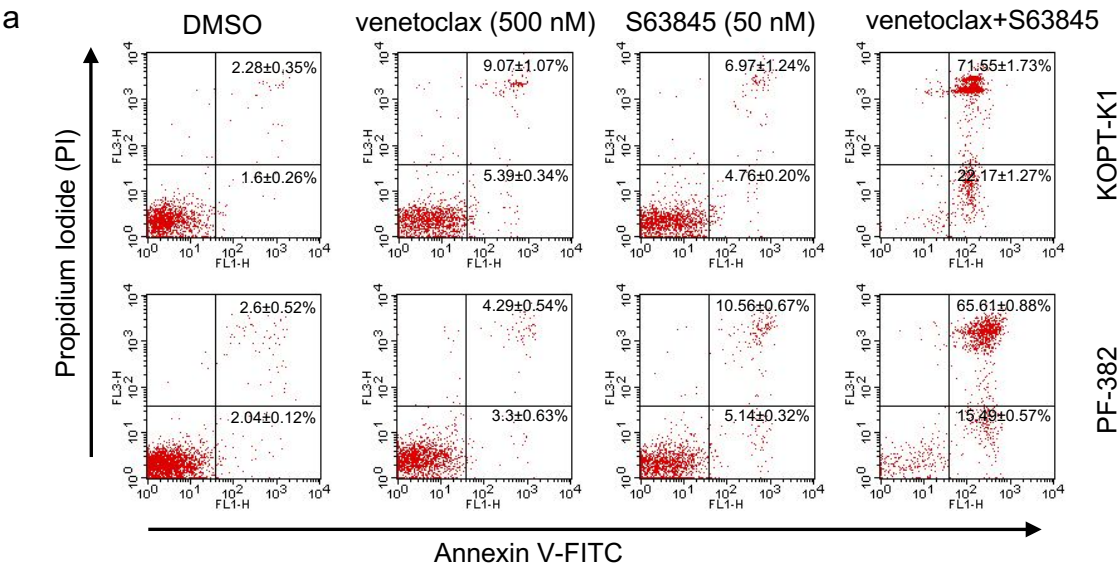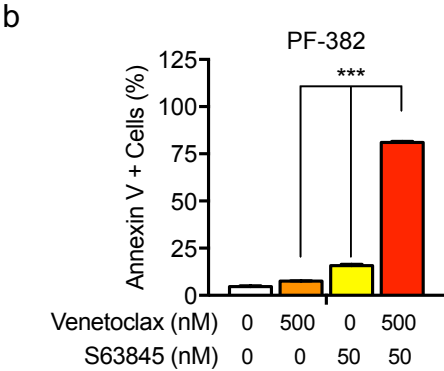

Supplement: Supplementary file 6 — Supplementary information Figure 5 [file 41375_2018_201_MOESM6_ESM.pdf]

Supplementary Figure 6

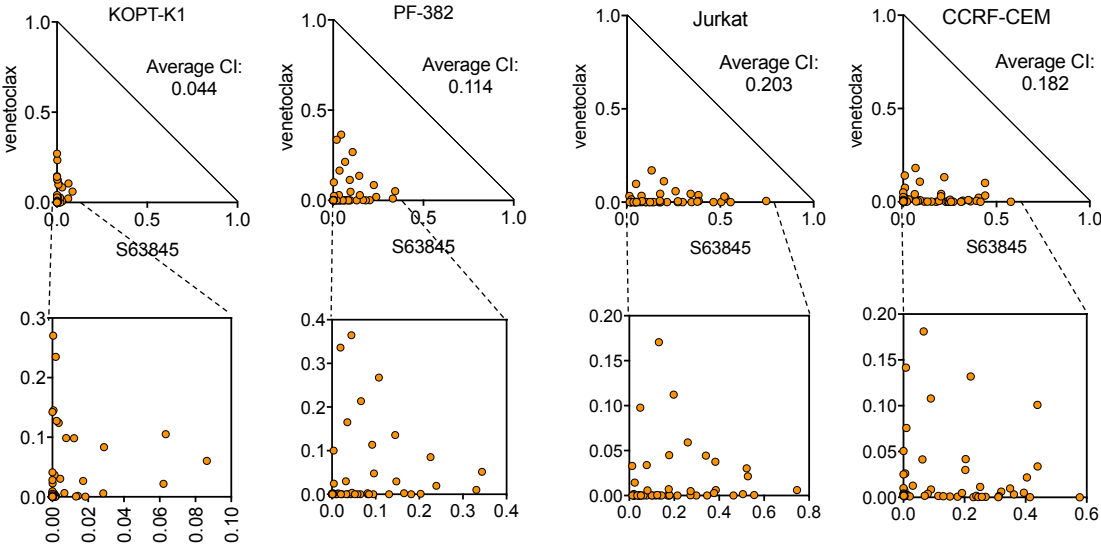

Supplement: Supplementary file 7 — Supplementary information Figure 6 [file 41375_2018_201_MOESM7_ESM.pdf]

## Supplementary Figure 7

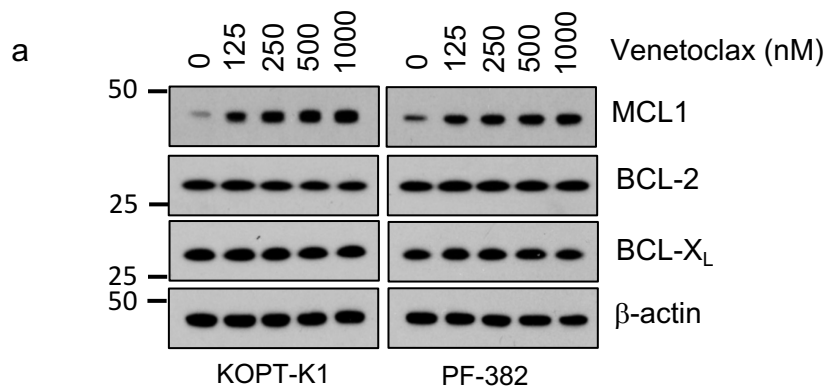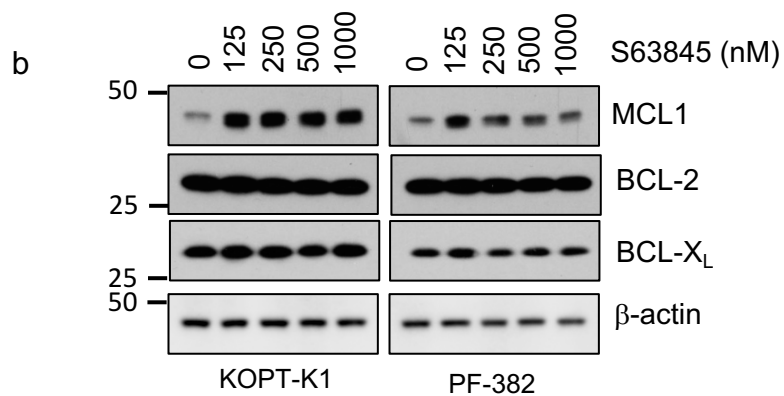

Supplement: Supplementary file 8 — Supplementary information Figure 7 [file 41375_2018_201_MOESM8_ESM.pdf]

Supplementary Figure 8

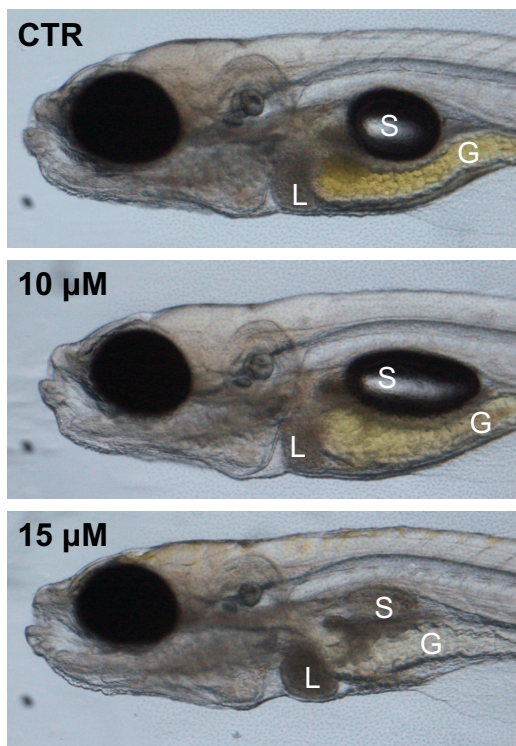

Supplement: Supplementary file 9 — Supplementary information Figure 8 [file 41375_2018_201_MOESM9_ESM.pdf]

Supplementary Figure 9

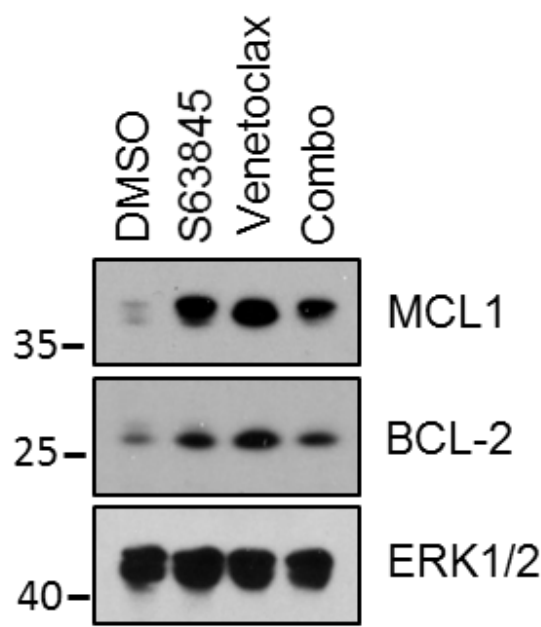

Supplement: Supplementary file 10 — Supplementary information Figure 9 [file 41375_2018_201_MOESM10_ESM.pdf]

Supplementary Figure 10

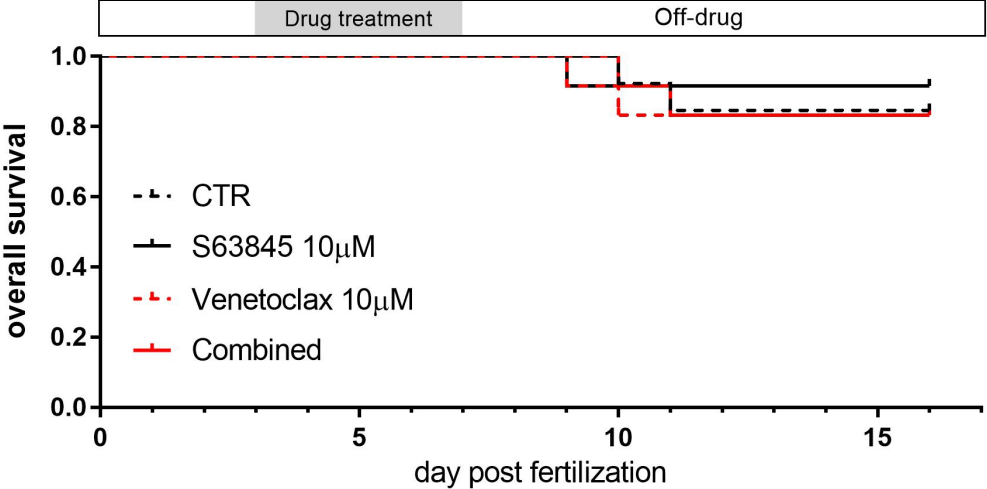

Supplement: Supplementary file 11 — Supplementary information Figure 10 [file 41375_2018_201_MOESM11_ESM.pdf]
